# QTL Mapping and Inheritance of Clubroot Resistance Genes Derived From *Brassica rapa* subsp. *rapifera* (ECD 02) Reveals Resistance Loci and Distorted Segregation Ratios in Two *F*_2_ Populations of Different Crosses

**DOI:** 10.3389/fpls.2020.00899

**Published:** 2020-07-03

**Authors:** Rudolph Fredua-Agyeman, Junye Jiang, Sheau-Fang Hwang, Stephen E. Strelkov

**Affiliations:** Department of Agricultural, Food and Nutritional Science, University of Alberta, Edmonton, AB, Canada

**Keywords:** *Plasmodiophora brassicae*, Chi-square test, segregation ratios, epistatic interaction, QTL mapping

## Abstract

In this study, *Brassica rapa* subsp. *rapifera* (ECD 02) which exhibits broad-spectrum resistance to many Canadian *Plasmodiophora brassicae* isolates was crossed with two clubroot-susceptible *B. rapa* accessions to produce two *F*_2_ populations. The *F*_2_ plants were screened against *P. brassicae* pathotypes 3H, 5X, and 5G. The Chi-square goodness of fit test showed that the vast majority (≈75%) of the crosses that produced the *F*_2_ populations showed segregation ratios of 9R:7S, 7R:9S, 13R:3S, 3R:13S, 5R:11S, 11R:5S, and 1R:15S. These were modifications of the 15R:1S ratio expected for the inheritance of two dominant major clubroot resistance (CR) genes from ECD 02. The distorted segregation ratios suggest that the two resistance genes are on different chromosomes and that two genes interact in an epistatic manner to confer resistance. Genotyping was conducted with 144 PCR-based markers in the two *F*_2_ populations. Linkage and QTL analysis with the polymorphic markers identified two QTLs on chromosome A03 to be associated with resistance to *P. brassicae* pathotypes 5X and 5G in Popl#1 while only the second QTL on chromosome A03 was associated with resistance to pathotypes 5X and 5G in Popl#2. The QTLs clustered in genomic regions on the A03 chromosome of *B. rapa* where the *CRa*/*CRb*^Kato^ gene(s) are mapped. In addition, the *Crr1* gene on the A08 chromosome of *B. rapa* was detected in the two *F*_2_ populations. Therefore, the phenotypic and molecular data confirm the existence of two CR genes in ECD 02. This is the first study that shows that major dominant genes in *Brassica* interact in a non-additive manner to confer resistance to different *P. brassicae* pathotypes.

**Key Message:** This study provides knowledge on the inheritance and type of gene action for clubroot resistance derived from *Brassica rapa* subsp. *rapifera* (ECD 02). The results indicated that duplicate recessive and recessive suppression epistatic interactions, digenic additivity and complementary gene action between the *CRa*/*CRb*^Kato^ gene(s) on the A03 and the *Crr1* gene on the A08 chromosome of *B. rapa* controlled clubroot resistance to *P. brassicae* pathotypes 3H, 5X and 5G.

## Introduction

Clubroot is a soil-borne disease of the Brassicaceae caused by the obligate parasite *Plasmodiophora brassicae*. Disease development is associated with the formation of large galls on the roots of susceptible plants, which interfere with water and nutrient uptake and lead to significant yield losses in *Brassica* crops (Hwang et al., 2012; Dixon, 2014). Yield losses of 20–100% have been reported worldwide including in Canada (Tewari et al., 2005; Rahman et al., 2014), China (Chai et al., 2014), and India (Bhattacharya et al., 2014). The clubroot pathogen survives as resting spores that can persist in the soil for many years (Dixon, 2009). Given the longevity of *P. brassicae* in infested soils and the significant economic value of *Brassica* crops, the management of clubroot has been a focus of agricultural researchers for decades. In recent years, clubroot has emerged as an important constraint to canola (*Brassica napus*; oilseed rape) production in western Canada, further increasing interest in this disease (Strelkov and Hwang, 2014).

While many strategies have been employed for clubroot control (Hwang et al., 2014), the planting of clubroot resistance (CR) canola cultivars is the most effective and environmentally friendly approach to manage the disease (Rahman et al., 2014). The identification of effective resistance is the first step in breeding for this trait, with *Brassica rapa* (2*n* = 20, AA) considered a superior source of dominant major clubroot resistance genes than *Brassica oleracea* (2*n* = 18, CC) (Toxopeus et al., 1986; Hirai, 2006; Piao et al., 2009). Over the past 20 years, at least 15 CR genes have been identified in *B. rapa*, including *CRa* (Matsumoto et al., 1998), *CRb* (Piao et al., 2004), *CRb*^Kato^ (Kato et al., 2012, 2013), *CRk* (Matsumoto et al., 2012), *Crr3* (Hirai et al., 2004; Saito et al., 2006), *Rcr1* (Chu et al., 2014; Yu et al., 2016), *CRc* (Sakamoto et al., 2008; Matsumoto et al., 2012), *Crr1*, *Crr2*, *Crr4* (Suwabe et al., 2003, 2006), *CRd* (Pang et al., 2018), *BraA.Cr.a*, *BraA.Cr.b*, *BraA.Cr.c* (Hirani et al., 2018), and *CrrA5* (Nguyen et al., 2018). Clubroot resistance genes from *B. rapa* have been introgressed into several European *B. napus* oilseed cultivars, including ‘Mendel’ and ‘Tosca’ (Frauen, 1999).

In Canada, different *Brasscia* genotypes have been used as resistance donors in the breeding of CR canola/oilseed rape (*B. napus*). Fredua-Agyeman and Rahman (2016) found that the *B. napus* cultivar ‘Mendel’ contained one dominant CR gene effective against pathotype 3H of *P. brassicae*, as defined on the Canadian Clubroot Differential Set (Strelkov et al., 2018). This was the dominant pathotype in Alberta, Canada (Strelkov et al., 2006, 2007), at least prior to the introduction of CR canola cultivars beginning in 2009 (Strelkov et al., 2018). However, the planting of CR canola in short rotations over large acreages led to the rapid development of new pathotypes of *P. brassicae* (Strelkov et al., 2016, 2018), and ‘Mendel’ resistance was eroded and is no longer a good choice for breeding cultivars resistant to the new pathotypes. Rutabaga (*Brassica napus* ssp. *napobrassica*) is another potential donor of clubroot resistance genes. The rutabaga cultivars ‘Brookfield’ and ‘Polycross’ possessed excellent resistance to Canadian *P. brassicae* pathotypes 2, 3, 5, 6, and 8 (Hasan and Rahman, 2011, 2016; Hasan and Rahman, 2013). The ratio of resistant (R) to susceptible (S) in the *F*_2_ generation derived from crossing both Brookfield and Polycross with susceptible *B. napus* lines was 3R:1S while segregation in the test cross family of the latter deviated from a 1R:1S ratio. This suggested that CR in Brookfield was controlled by a single dominant gene while resistance in Polycross was more complex (Hasan and Rahman, 2011).

With the recent emergence of new pathotypes of *P. brassicae* and their ability to overcome clubroot resistance in most CR canola cultivars, additional sources of resistance are needed. The *CRa* resistance gene was first detected in the European Clubroot Differential (ECD) 02 (*B. rapa* ssp. *rapifera* line AAbbCC) (Buczacki et al., 1975; Matsumoto et al., 1998). Zhang et al. (2016) reported one marker (i3e4) that was tightly linked to *CRa*. While ECD 02 appears to be resistant to all *P. brassicae* pathotypes identified in Canada (Strelkov et al., 2006, 2018; Leboldus et al., 2012), and was used as a resistance source in studies from Japan (Hayashida et al., 2008; Sakamoto et al., 2008), its application in clubroot resistance breeding in Canada has not yet been reported. The objectives of the current study were to introgress clubroot resistance from ECD 02 (male parent) into two susceptible female *B. rapa* genotypes CR 2599 and CR 1505, to evaluate the genetic basis of resistance to three isolates of *P. brassicae* representing different pathotypes and identify molecular markers associated with resistance to the new pathotypes.

## Materials and Methods

### Development of Mapping Populations

The parent *B. rapa* subsp. *rapifera* line AAbbCC (ECD 02) was resistant to all 17 *P*. *brassicae* pathotypes identified in Canada (Strelkov et al., 2018). In contrast, *B. rapa* accessions CR 2599 and CR 1505 (‘Emma’) were susceptible to these same pathotypes (Fredua-Agyeman et al., 2019) and served as the susceptible parents. ECD 02 is a winter-type while the two susceptible parents are spring-type.

To produce the F_1_ plants, genetic crosses were carried out between June 2016 and January 2017 by emasculation followed by hand-pollination, with the plants kept in a growth chamber maintained under an 18 h photoperiod and temperatures of 21/18°C (day/night). Vernalization of ECD 02 was carried out as described by Fredua-Agyeman et al. (2019). The susceptible parents were seeded much later to ensure that they flowered around the same time as ECD 02.

Seeding, vernalization and the self-pollination of single F_1_ plants to produce *F*_2_ seeds were carried out in a growth chamber, cold room and greenhouses, respectively, at the Crop Diversification Centre North, Alberta Agriculture and Forestry, Edmonton, Alberta from March 2017 to June 2018. The ECD 02 × CR 2599 and ECD 02 × CR 1505 derived *F*_2_ populations will be designated here as Popl#1 and Popl#2, respectively, for convenience.

### Pathogen Material

Twenty-two *P. brassicae* field and single-spore isolates representing 17 unique pathotypes were used to screen 14–24 seedlings of ECD 02. The pathotypes (isolates indicated in parentheses) included: pathotype 2B (F183-14), 2F (SACAN-ss3), 3A (F3-14), 3D (F1-14), 3H (SACAN-ss1 and CDCN#4), 3O (F381-16), 5C (F175-14), 5G (CDCS and CDCN#6), 5I (ORCA-ss4 and CDCN#2), 5K (F10-15), 5L (F-360-13), 5X (LG-1, LG-2, and LG-3), 6M (AbtJE-ss1), 8E (F187-14), 8J (F12-15), 8N (ORCA-ss2), and 8P (UoA#37) (Xue et al., 2008; Strelkov et al., 2016, 2018). Pathotype designations follow the Canadian Clubroot Differential (CCD) Set (Strelkov et al., 2018).

The number of isolates used to screen the F_1_ seedlings depended on the number of seeds obtained from each cross. Five single-spore isolates (representing pathotypes 2F, 3H, 5I, 6M, and 8N) (Xue et al., 2008) and four field isolates [representing pathotypes 2B, 5X (LG-1), 5G (CDCS), and 8J] (Strelkov et al., 2016, 2018) were used to screen 25 F_1_ plants of the cross ECD 02 × CR 2599 (Popl#1). Two isolates [pathotypes 5X (LG-1) and 5G (CDCS)] were used to screen two F_1_ plants of the cross ECD 02 × CR1505 (Popl#2).

*F*_2_ plants of the two populations were screened with pathotypes 3H (predominant in Alberta and represented by the single-spore isolate SACAN-ss1), 5X (the first to overcome resistance in CR canola and represented by the field isolate LG-1; Strelkov et al., 2016), and 5G (represented by isolate CDCS, one of the most virulent isolates used in an earlier study; Fredua-Agyeman et al., 2019). Thus, segregating ratios in the two *F*_2_ populations (Popl#1 and Popl#2) × pathotypes 3H (SACAN-ss1), 5X (LG-1), and 5G (CDCS) (i.e., six combinations) were examined.

### Inoculum Preparation

Isolates of *P. brassicae* were stored as galled roots at −20°C until needed. To prepare resting spore inoculum, the galls were homogenized in sterile distilled water in a Waring LB10G blender (Cole-Parmer) following Fredua-Agyeman et al. (2018). The resulting slurry was passed through eight layers of cheesecloth into a beaker to remove plant debris and other detritus, and the filtrate was collected. The resting spore concentration was estimated using a hemocytometer and adjusted to 1 × 10^8^ spores/mL with sterile distilled water. Inoculum was kept at 4°C and used within 24 h of preparation.

### Clubroot Assays

Seedlings were geminated in Petri dishes (100 mm × 15 mm) on moistened Whatman no. 1 filter paper for 7 days at room temperature and a 12 h photoperiod. Inoculations were carried using the root-dip method (Nieuwhof and Wiering, 1961; Strelkov et al., 2006), with additional inoculum added by the pipette method (Lamers and Toxopeus, 1977 as cited by Voorrips and Visser, 1993). Briefly, the rootlets were dipped into the pathogen resting spore suspension for about 10–20 s and then planted in 8 × 4 flat trays filled with Sunshine Mix #4 Aggregate Plus (Sun Gro Horticulture, Seba Beach, AB, Canada) potting medium. This was followed by pipetting 1 ml of inoculum and dispensing into the soil surrounding each seedling.

The seedlings were transferred to a greenhouse maintained at 20–25/15–18°C day/night with a 16 h photoperiod, and watered daily with slightly acidified water (pH ≈ 5.5–6.5, adjusted with HCL). Beginning at 3 weeks after inoculation, the plants were fertilized once a week with 20 N: 20 P: 20 K Classic Fertilizer with micronutrients (Plant Products Brampton, ON, Canada).

Eight weeks after inoculation, the plants were gently removed from the potting medium, washed in water, and assessed for clubroot severity on a 0–3 scale, where: 0 = no galls; 1 = a few small or bead-sized galls on <1/3 of the roots; 2 = medium galls on 1/3–2/3 of the roots, and 3 = large galls on >2/3 of the roots (Kuginuki et al., 1999; Xue et al., 2008). The susceptible *B*. *napus* ‘Westar’ was included as a positive control in all of the assays.

The segregating ratios in the two *F*_2_ populations (Popl#1 and Popl#2) × pathotypes 3H (SACAN-ss1), 5X (LG-1) and 5G (CDCS) (i.e., six combinations) were examined.

### DNA Extraction

Three hundred sixty-eight leaves were collected from 46 *F*_2_ individuals resistant (disease score = 0) and 46 *F*_2_ individuals susceptible (disease score = 3) to each of pathotypes 5X and 5G of Popl#1 and Popl#2. Genomic DNA was extracted from the leaves of the parents and the 368 *F*_2_ individuals using the cetyltrimethyl ammonium bromide (CTAB) method (Sambrook and Russell, 2001). The DNA concentration was quantified with a ND-2000c spectrophotometer (NanoDrop Technologies, Inc., Wilmington, DE, United States) and the template DNA diluted to 20–25 ng/μL for PCR.

No molecular work was carried out on *F*_2_ plants inoculated with pathotype 3H (for the two populations) because almost all previous genetic mapping studies in Canada have utilized this pathotype. Therefore, molecular analyses were completed on four of the six plant population/*P. brassicae* pathotype combinations.

### PCR and SSR Genotyping

PCR amplification was carried out in a 12 μL reaction volume containing 2.5 μL of 5 × Taq buffer, 1.0 μL of 25 mM MgCl_2_, 0.25 μL of 10 mM dNTPs mix, 0.25 μL of 25 nM of each primer, 0.25 μL of 25 nM of fluorescently labeled M13 primer, 1.0 μL of 20–25 ng DNA template, and 1.25 U of Taq polymerase (Promega, Madison, WI, United States). The PCR cycling conditions consisted of an initial denaturation at 95°C for 5 min, followed by 35 cycles at 95°C for 1 min, 58°C for 1 min and 72°C for 1.5 min, and a final elongation step at 72°C for 10 min. Aliquots of the PCR products were analyzed on an ABI PRISM 3730 × l DNA analyzer (Applied Biosystems). Amplified products from resistant and susceptible plants which differed by >200 bp were separated on 3% agarose gels. The gels were stained with SYBR Safe DNA Gel Stain (Thermo Fisher Scientific, Carlsbad, CA, United States) and visualized with a Typhoon FLA 9500 Variable Mode Laser Scanner Image Analyzer (GE Healthcare Life Sciences, Mississauga, ON, Canada).

Bulk segregant analysis as described by Michelmore et al. (1991) was carried using 144 PCR-based markers with DNA of the parents and two resistant and two susceptible DNA bulks from each of the two *F*_2_ populations (Popl#1 and Pop#2). The markers included 13 markers spanning the A03 chromosome of *B. rapa*, 65 markers linked to six previously reported clubroot resistant genes: *CRk*, *Crr3*, *CRb*, *CRb*^Kato^, *CRa*, and *Crr1*, as well as 66 markers designed in this study. Of the 66 markers, 50 were SSR markers designed from the identified QTL regions while the remaining 16 were designed based on the *CRa* gene sequence of Ueno et al. (2012). The polymorphic markers were used to genotype the parents as well as the 384 *F*_2_ individuals of Popl#1 and Pop#2, and a genotype matrix was constructed for pathotypes 5X and 5G for Popl#1 and Pop#2.

### Map Construction and QTLs Analysis

Genetic linkage analysis was performed for each of the four combinations using MAPMAKER/EXP 3.0 (Lincoln et al., 1992). The logarithm of odds (LOD) score was set for a minimum of 3.0 and a recombination fraction (O) of 0.40. The Kosambi map function was used to convert the recombination frequencies into genetic distances (in centimorgans, cM) (Kosambi, 1944). Genetic linkage maps were constructed using MapChart v. 2.32 (Voorrips, 2002). Inclusive Composite Interval Mapping-Additive (ICIM-ADD) and Single Marker Analysis (SMA) methods were performed based on stepwise regression of simultaneous consideration of all markers using the IciMapping software V4.1 with 1 cM walking speed, 1000 permutations and *P* < 0.05 (Meng et al., 2015). To confirm a QTL, Composite Interval Mapping (CIM) and SMA were performed at the same mapping parameters as the above (Churchill and Doerge, 1994) using WinQTL Cartographer software v2.5 (Wang et al., 2011). A definitive QTL was declared if peaks with LOD score ≥ 3.0 were identified at the same position by both the IciMapping software V4.1 and WinQTL Cartographer software V2.5. Smaller peaks in close proximity to major peaks were regarded as artifacts. The QTLs identified in this study were named using a modified gene nomenclature system for the *Brassica* genus proposed by Østergaard and King (2008). The QTL name was in the order genus (1 letter), species (2 letters), genome (1 letter), chromosome number (1 letter), pathotype name (3 letters), closest published gene(s) (3–6 letters), and QTL number (2 letters). The additive effects of individual QTLs and epistatic interaction (QTL × QTL) were estimated as the percentage of phenotypic variation explained (PVE) by the IciMapping software V.4.1 using the ICIM-ADD and ICIM-EPI methods, respectively.

### Statistical Analysis

To test for the inheritance of clubroot resistance in the two *F*_2_ populations, the phenotypic data from different crosses of the same parents were subjected to Chi-square (χ^2^) tests of homogeneity. In addition, individual data from the different crosses found to be uniform were pooled. The resulting data for each population were subjected to χ^2^ goodness-of-fit for different segregation ratios using SAS v. 9.4 (SAS Institute, United States).

## Results

### Clubroot Assays, Genetic Crosses and Development of *F*_2_ Mapping Populations

In total, 98% (428 of 435) of ECD 02 plants inoculated with the 22 isolates of *P. brassicae* (representing 17 unique pathotypes) were completely free of clubroot symptoms (disease score = 0) 8 weeks after inoculation. Sixteen of the 22 isolates did not cause any symptoms at all (disease score = 0) on any plants, while very mild galling (disease score = 1) was observed on a single plant in response to inoculation with each of pathotypes 2B (F183-14), 5I (ORCA-ss4), 5X (F-359-13), 8N (ORCA-ss2), and 8J (F12-15). Two plants of ECD 02 inoculated with pathotype 5G (CDCS, Newell) developed moderate galling (disease score = 2).

Eleven plants of ECD 02 showing absolute resistance (disease score = 0) to pathotype 5G were used as donor parents in crosses with the two susceptible parents CR 2599 and CR 1509. The F_1_ plants produced from the two crosses were winter-types and required vernalization to induce flowering. Approximately 76% (25 of 33) and 25% (2 of 8) of the crosses carried out between ECD 02 and CR 2599 and CR 1509, respectively, resulted in siliques. Of the successful crosses, 24% (6 of 25) and 50% (1 of 2) produced 2–30 good quality rounded seeds per silique. All 27 of the F_1_ plants screened with *P. brassicae* (2–9 pathotypes) were resistant to the pathogen (23 disease score = 0, 4 disease score = 1) (Supplementary Table S1). The 23 F_1_ plants with a disease score = 0 were used as donors of clubroot resistance genes for the various crosses.

**FIGURE 1:**
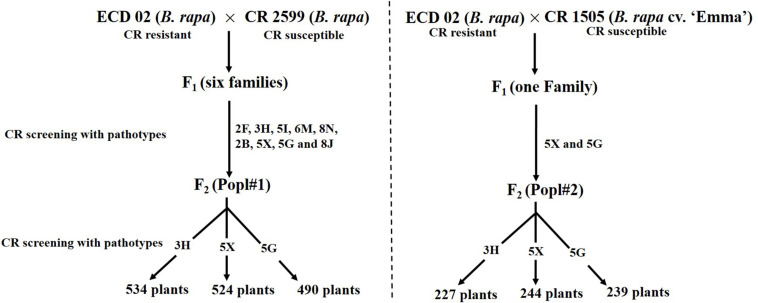
Development of *F*_2_ mapping population for the study of the inheritance pattern of clubroot resistance introgressed from ECD 02.

**FIGURE 2:**
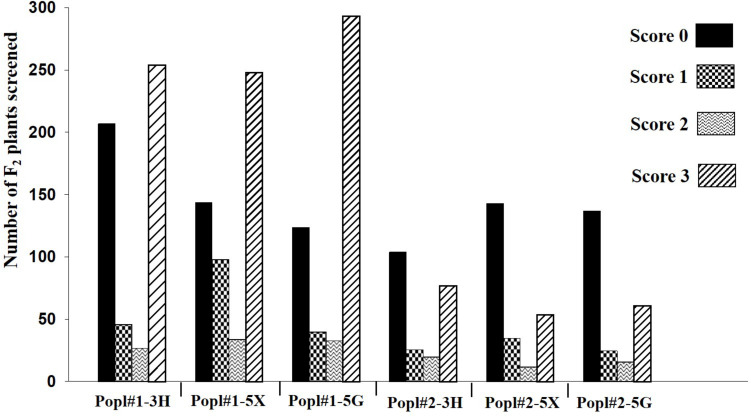
Frequency distribution of disease scores in two *F*_2_ populations (Popl#1 and Popl#2) derived from ECD 02 × CR 2599 and ECD 02 × CR 1505 (‘Emma’) to three *Plasmodiophora brassicae* pathotypes 3H, 5X, and 5G.

The development of two *F*_2_ mapping populations from the crosses between ECD 02 and the two susceptible parents is summarized in Figure 1. One thousand five hundred and forty-eight and 710 *F*_2_ individuals derived from 6 and 1 clubroot resistant F_1_ families from Popl#1 and Popl#2, respectively, were evaluated for resistance to the *P. brassicae* pathotypes 3H (SACAN-ss1), 5X (LG-1), and 5G (CDCS). The frequency distribution of disease scores to the three pathotypes in the two *F*_2_ populations is presented in Figure 2. All six distributions were bimodal, suggesting oligogenic or polygenic resistance to clubroot in the two *F*_2_ populations.

### Phenotypic Variation in *F*_2_ Mapping Subpopulations

The χ^2^ tests of homogeneity indicated that the phenotypic data from the 534 *F*_2_ plants of Popl#1 inoculated with pathotype 3H and the 524 *F*_2_ plants inoculated with pathotype 5X were significantly different (*P* < 0.00001) and hence could not be pooled (Supplementary Tables S2, S3). In contrast, no significant differences were found in the phenotypic data from the 490 *F*_2_ plants of Popl#1 inoculated with pathotype 5G (Supplementary Table S4).

In the case of Popl#2, the 227, 244, and 239 *F*_2_ plants inoculated with pathotypes 3H, 5X, and 5G, respectively, were produced by self-pollination of a single F_1_ family from the cross ECD 02 × CR 1505 and hence, no χ^2^ tests of homogeneity were conducted. Instead, the *F*_2_ data for Popl#2 were tested directly for χ^2^ goodness-of-fit for different segregation ratios.

### Inheritance Pattern of Clubroot Resistance Derived From ECD 02

The segregation analysis was carried out in two ways. The first considered only those plants with a disease score = 0 as resistant (R), and all others as susceptible (S) (Tables 1, 2). The second considered plants with disease scores = 0 or 1 as R, and those with disease scores = 2 or 3 as S (Tables 1, 2). The Chi-square goodness of fit test showed that the segregation of clubroot resistance in the *F*_2_ populations largely deviated from the expected Mendelian segregation ratios of 3:1, 15:1, and 63:1 for resistance controlled by a single, two or three dominant genes, respectively. Instead, deviations from the normal ratios, such as those observed in the event of non-allelic or linked interactions, were obtained.

**TABLE 1:** Segregation ratios for ECD 02 × CR 2599 derived *F*_2_ population for resistance to clubroot under greenhouse conditions.

Popl#/Fam	Pathotype^*φ*^	Total no. of *F*_2_ (Score = No. plants)	Tested ratio	DF	Observed (disease score)^1^		Test of statistics^1^		Observed (disease score)^2^		Test of statistics^2^	
								
					*R* (0)	*S* (1 + 2 + 3)	χ^2^	Prob	*R* (0 + 1)	*S* (2 + 3)	χ^2^	Prob
Popl#1 Fam 1	3H (SACAN-ss1)	34 (Score 0 = 10) (Score 1 = 4) (Score 2 = 2) (Score 3 = 18)	3R:1S	1	10	24	37.7	<0.00001	14	20	20.7	<0.00001
			1R:3S	1			0.4	0.5525*			4.7	0.0294
			9R:7S	1			10.0	0.0016			3.1	0.0764*
			7R:9S	1			2.8	0.0919*			0.1	0.7623*
			5R:11S	1			0.1	0.8171*			1.6	0.2118*
			11R:5S	1			24.5	<0.00001			12.0	0.0005
			13R:3S	1			60.0	<0.00001			35.8	<0.00001
			3R:13S	1			2.5	0.1112*			11.2	0.0008
			15R:1S	1			240.2	<0.00001			160.4	<0.00001
			1R:15S	1			31.1	<0.00001			70.8	<0.00001
			63R:1S	1			1053.2	<0.00001			724.8	<0.00001
			1R:63S	1			171.4	<0.00001			346.9	<0.00001
Popl#1 Fam 2	3H (SACAN-ss1)	68 (Score 0 = 39) (Score 1 = 18) (Score 2 = 2) (Score 3 = 9)	3R:1S	1	39	29	11.3	0.0008	57	11	2.8	0.0929*
			1R:3S	1			38.0	<0.00001			125.5	<0.00001
			9R:7S	1			0.03	0.8545*			21.0	<0.00001
			7R:9S	1			5.1	0.0237			44.4	<0.00001
			5R:11S	1			21.6	<0.00001			87.5	<0.00001
			11R:5S	1			4.1	0.0426			7.2	0.0073
			13R:3S	1			25.5	<0.00001			0.3	0.5866*
			3R:13S	1			66.5	<0.00001			189.0	<0.00001
			15R:1S	1			153.7	<0.00001			11.4	0.0007
			1R:15S	1			303.1	<0.00001			698.4	<0.00001
			63R:1S	1			746.3	<0.00001			94.4	<0.00001
			1R:63S	1			1376.1	<0.00001			2991.7	<0.00001
Popl#1 Fam 3	3H (SACAN-ss1)	151 (Score 0 = 34) (Score 1 = 4) (Score 2 = 11) (Score 3 = 102)	3R:1S	1	34	117	221.8	<0.00001	38	113	200.0	<0.00001
			1R:3S	1			0.5	0.4810*			0.002	0.9625*
			9R:7S	1			69.8	<0.00001			59.3	<0.00001
			7R:9S	1			27.7	<0.00001			21.2	<0.00001
			5R:11S	1			5.4	0.0206			2.6	0.1067*
			11R:5S	1			150.2	<0.00001			133.5	<0.00001
			13R:3S	1			341.9	<0.00001			311.8	<0.00001
			3R:13S	1			1.4	0.2357*			4.1	0.0434
			15R:1S	1			1307.7	<0.00001			1212.2	<0.00001
			1R:15S	1			68.2	<0.00001			92.2	<0.00001
			63R:1S	1			5658.7	<0.00001			5270.7	<0.00001
			1R:63S	1			431.1	<0.00001			546.9	<0.00001
Popl#1 Fam 4	3H (SACAN-ss1)	176 (Score 0 = 65) (Score 1 = 12) (Score 2 = 9) (Score 3 = 90)	3R:1S	1	65	111	136.0	<0.00001	77	99	91.7	<0.00001
			1R:3S	1			13.4	0.0003			33.0	<0.00001
			9R:7S	1			26.7	<0.00001			11.2	0.0008
			7R:9S	1			3.3	0.0682*			0.00	1.00*
			5R:11S	1			2.6	0.1039*			12.8	0.0003
			11R:5S	1			82.9	<0.00001			51.2	<0.00001
			13R:3S	1			226.9	<0.00001			162.5	<0.00001
			3R:13S	1			38.2	<0.00001			72.2	<0.00001
			15R:1S	1			969.7	<0.00001			750.9	<0.00001
			1R:15S	1			282.8	<0.00001			422.4	<0.00001
			63R:1S	1			4328.8	<0.00001			3422.2	<0.00001
			1R:63S	1			1431.5	<0.00001			2036.6	<0.00001
Popl#1 Fam 5	3H (SACAN-ss1)	50 (Score 0 = 39) (Score 1 = 1) (Score 2 = 0) (Score 3 = 10)	3R:1S	1	39	11	0.2	0.6242*	40	10	0.7	0.4142*
			1R:3S	1			74.9	<0.00001			80.7	<0.00001
			9R:7S	1			9.6	0.0019			11.5	0.0007
			7R:9S	1			23.8	<0.00001			26.7	<0.00001
			5R:11S	1			50.9	<0.00001			55.3	<0.00001
			11R:5S	1			2.0	0.1582*			2.9	0.0861*
			13R:3S	1			0.3	0.5560*			0.1	0.8208*
			3R:13S	1			115.2	<0.00001			123.1	<0.00001
			15R:1S	1			21.2	<0.00001			16.1	0.00006
			1R:15S	1			439.3	<0.00001			464.1	<0.00001
			63R:1S	1			135.8	<0.00001			110.5	<0.00001
			1R:63S	1			1899.3	<0.00001			2000.0	<0.00001
Popl#1 Fam 6	3H (SACAN-ss1)	55 (Score 0 = 20) (Score 1 = 7) (Score 2 = 3) (Score 3 = 25)	3R:1S	1	20	35	43.8	<0.00001	27	28	19.7	<0.00001
			1R:3S	1			3.8	0.0516*			17.0	0.00004
			9R:7S	1			8.8	0.0030			1.1	0.2845*
			7R:9S	1			1.2	0.2695*			0.6	0.4246*
			5R:11S	1			0.7	0.4133*			8.1	0.0043
			11R:5S	1			26.9	<0.00001			9.9	0.0017
			13R:3S	1			72.7	<0.00001			37.3	<0.00001
			3R:13S	1			11.2	0.0008			33.2	<0.00001
			15R:1S	1			309.1	<0.00001			187.2	<0.00001
			1R:15S	1			85.1	<0.00001			172.3	<0.00001
			63R:1S	1			1377.8	<0.00001			870.8	<0.00001
			1R:63S	1			433.1	<0.00001			807.8	<0.00001
Popl#1 Fam 1	5X (LG-1)	76 (Score 0 = 25) (Score 1 = 19) (Score 2 = 4) (Score 3 = 28)	3R:1S	1	25	51	71.9	<0.00001	44	32	11.9	0.0006
			1R:3S	1			2.5	0.1120*			43.9	<0.00001
			9R:7S	1			16.8	0.00004			0.1	0.7726*
			7R:9S	1			3.6	0.0564*			6.2	0.0129
			5R:11S	1			0.1	0.7571*			25.1	<0.00001
			11R:5S	1			45.5	<0.00001			4.2	0.0412
			13R:3S	1			116.6	<0.00001			27.2	<0.00001
			3R:13S	1			10.0	0.0016			76.4	<0.00001
			15R:1S	1			480.4	<0.00001			166.8	<0.00001
			1R:15S	1			92.1	<0.00001			346.0	<0.00001
			63R:1S	1			2122.7	<0.00001			812.2	<0.00001
			1R:63S	1			485.1	<0.00001			1568.0	<0.00001
Popl#1 Fam 2	5X (LG-1)	47 (Score 0 = 4) (Score 1 = 10) (Score 2 = 3) (Score 3 = 30)	3R:1S	1	4	43	110.8	<0.00001	14	33	51.2	<0.00001
			1R:3S	1			6.8	0.0090			0.6	0.4485*
			9R:7S	1			43.5	<0.00001			13.4	0.0003
			7R:9S	1			23.7	<0.00001			3.7	0.0537*
			5R:11S	1			11.3	0.0008			0.05	0.8287*
			11R:5S	1			79.4	<0.00001			33.2	<0.00001
			13R:3S	1			163.2	<0.00001			81.7	<0.00001
			3R:13S	1			3.2	0.0721*			3.8	0.0525*
			15R:1S	1			582.8	<0.00001			328.2	<0.00001
			1R:15S	1			0.4	0.5220*			44.4	<0.00001
			63R:1S	1			2471.1	<0.00001			1440.1	<0.00001
			1R:63S	1			14.8	0.0001			243.4	<0.00001
Popl#1 Fam 3	5X (LG-1)	142 (Score 0 = 59) (Score 1 = 27) (Score 2 = 6) (Score 3 = 50)	3R:1S	1	59	83	84.7	<0.00001	86	56	15.8	0.00007
			1R:3S	1			20.7	<0.00001			95.8	<0.00001
			9R:7S	1			12.5	0.0004			1.1	0.3001*
			7R:9S	1			0.3	0.5971*			16.3	0.00005
			5R:11S	1			7.0	0.0081			56.8	<0.00001
			11R:5S	1			48.9	<0.00001			4.4	0.0353
			13R:3S	1			146.9	<0.00001			39.9	<0.00001
			3R:13S	1			48.5	<0.00001			163.0	<0.00001
			15R:1S	1			660.4	<0.00001			266.9	<0.00001
			1R:15S	1			302.0	<0.00001			714.9	<0.00001
			63R:1S	1			2987.8	<0.00001			1324.3	<0.00001
			1R:63S	1			1476.2	<0.00001			3213.8	<0.00001
Popl#1 Fam 4	5X (LG-1)	163 (Score 0 = 47) (Score 1 = 30) (Score 2 = 13) (Score 3 = 73)	3R:1S	1	47	116	185.3	<0.00001	77	86	67.0	<0.00001
			1R:3S	1			1.3	0.2582*			43.0	<0.00001
			9R:7S	1			49.8	<0.00001			5.4	0.0204
			7R:9S	1			14.7	0.0001			0.8	0.3692*
			5R:11S	1			0.4	0.5058*			19.4	0.00001
			11R:5S	1			120.9	<0.00001			35.1	<0.00001
			13R:3S	1			294.0	<0.00001			123.8	<0.00001
			3R:13S	1			10.9	0.0010			86.8	<0.00001
			15R:1S	1			1172.3	<0.00001			601.8	<0.00001
			1R:15S	1			141.9	<0.00001			467.4	<0.00001
			63R:1S	1			5134.1	<0.00001			2777.9	<0.00001
			1R:63S	1			788.2	<0.00001			2211.0	<0.00001
Popl#1 Fam 5	5X (LG-1)	56 (Score 0 = 2) (Score 1 = 7) (Score 2 = 7) (Score 3 = 40)	3R:1S	1	2	54	152.4	<0.00001	9	47	103.7	<0.00001
			1R:3S	1			13.7	0.0002			2.4	0.1228*
			9R:7S	1			63.1	<0.00001			36.7	<0.00001
			7R:9S	1			36.7	<0.00001			17.4	0.00003
			5R:11S	1			20.0	<0.00001			6.0	0.0143
			11R:5S	1			110.7	<0.00001			72.3	<0.00001
			13R:3S	1			221.8	<0.00001			156.2	<0.00001
			3R:13S	1			8.5	0.0036			0.3	0.6076*
			15R:1S	1			777.2	<0.00001			576.7	<0.00001
			1R:15S	1			0.7	0.4076*			9.2	0.0024
			63R:1S	1			3276.6	<0.00001			2470.0	<0.00001
			1R:63S	1			1.5	0.2254*			76.6	<0.00001
Popl#1Fam 6	5X(LG-1)	40 (Score 0 = 7) (Score 1 = 5) (Score 2 = 1) (Score 3 = 27)	3R:1S	1	7	33	70.5	<0.00001	12	28	43.2	<0.00001
			1R:3S	1			1.2	0.2733*			0.5	0.4652*
			9R:7S	1			24.4	<0.00001			11.2	0.0008
			7R:9S	1			11.2	0.0008			3.1	0.0796*
			5R:11S	1			3.5	0.0606*			0.03	0.8646*
			11R:5S	1			48.9	<0.00001			28.0	<0.00001
			13R:3S	1			106.7	<0.00001			69.0	<0.00001
			3R:13S	1			0.04	0.8395*			3.3	0.0683*
			15R:1S	1			396.9	<0.00001			277.4	<0.00001
			1R:15S	1			8.6	0.0033			38.5	<0.00001
			63R:1S	1			1703.6	<0.00001			1218.1	<0.00001
			1R:63S	1			66.1	<0.00001			210.3	<0.00001
Popl#1Pooled	5G(CDCS)	490 (Score 0 = 124) (Score 1 = 40) (Score 2 = 33) (Score 3 = 293)	3R:1S	1	124	366	645.4	<0.00001	164	326	450.7	<0.00001
			1R:3S	1			0.02	0.8756*			18.7	0.00001
			9R:7S	1			190.7	<0.00001			103.3	<0.00001
			7R:9S	1			67.7	<0.00001			21.0	<0.00001
			5R:11S	1			8.1	0.0045			1.1	0.2892*
			11R:5S	1			430.5	<0.00001			283.9	<0.00001
			13R:3S	1			1006.6	<0.00001			734.3	<0.00001
			3R:13S	1			13.8	0.0002			69.7	<0.00001
			15R:1S	1			3917.5	<0.00001			3038.8	<0.00001
			1R:15S	1			303.7	<0.00001			619.6	<0.00001
			63R:1S	1			17038.2	<0.00001			13446.7	<0.00001
			1R:63S	1			1796.0	<0.00001			3243.3	<0.00001

*^∗^Observed segregation ratio not significantly different from the tested ratio at P ≤ 0.05. ^ϕ^Pathotype 3H (SACAN-ss1) is a single spore isolate (Xue et al., 2008) while isolates represented by pathotypes 5X (LG-1) and 5G (CDCS) are field isolates (Strelkov et al., 2016, 2018). ^1^Plants with disease score = 0 as R and those with disease scores 1, 2 and 3 as S. ^2^ Plants with disease scores = 0 and 1 as R and those with disease scores 2 and 3 as S.*

**TABLE 2:** Segregation ratios for ECD 02 × CR 1505 derived *F*_2_ population for resistance to clubroot under greenhouse conditions.

Popl/Fam	Pathotype^*φ*^	Total no. of *F*_2_ (Score = no. plants)	Tested ratio	DF	Observed (disease score)^1^		Test of statistics^1^		Observed (disease score)^2^		Test of statistics^2^	
					*R* (0)	*S* (1 + 2 + 3)	χ^2^	Prob	*R* (0 + 1)	*S* (2 + 3)	χ^2^	Prob
Popl#2 Fam 1	3H (SACAN-ss1)	227 (Score 0 = 104) (Score 1 = 26) (Score 2 = 20) (Score 3 = 77)	3R:1S	1	104	123	103.1	<0.00001	130	97	38.1	<0.00001
			1R:3S	1			52.5	<0.00001			126.1	<0.00001
			9R:7S	1			10.0	0.0015			0.1	0.7570*
			7R:9S	1			0.4	0.5306*			16.9	0.00004
			5R:11S	1			22.4	<0.00001			71.5	<0.00001
			11R:5S	1			55.6	<0.00001			13.9	0.0002
			13R:3S	1			187.1	<0.00001			85.7	<0.00001
			3R:13S	1			109.1	<0.00001			221.1	<0.00001
			15R:1S	1			890.2	<0.00001			515.6	<0.00001
			1R:15S	1			606.5	<0.00001			1008.4	<0.00001
			63R:1S	1			4086.8	<0.00001			2501.4	<0.00001
			1R:63S	1			2890.2	<0.00001			4579.9	<0.00001
Popl#2Fam 1	5X(LG-1)	244 (Score 0 = 143) (Score 1 = 35) (Score 2 = 12) (Score 3 = 54)	3R:1S	1	143	101	35.0	<0.00001	178	66	0.5	0.4598*
			1R:3S	1			147.0	<0.00001			299.2	<0.00001
			9R:7S	1			0.6	0.4581*			27.7	<0.00001
			7R:9S	1			21.9	<0.00001			84.5	<0.00001
			5R:11S	1			85.0	<0.00001			197.5	<0.00001
			11R:5S	1			11.7	0.0006			2.0	0.1569*
			13R:3S	1			82.1	<0.00001			11.0	0.0009
			3R:13S	1			254.4	<0.00001			470.5	<0.00001
			15R:1S	1			514.3	<0.00001			180.1	<0.00001
			1R:15S	1			1141.5	<0.00001			1852.7	<0.00001
			63R:1S	1			2516.8	<0.00001			1030.5	<0.00001
			1R:63S	1			5162.1	<0.00001			8084.7	<0.00001
Popl#2Fam 1	5G(CDCS)	239 (Score 0 = 137) (Score 1 = 25) (Score 2 = 16) (Score 3 = 61)	3R:1S	1	137	102	39.8	<0.00001	162	77	6.6	0.0010
			1R:3S	1			133.2	<0.00001			233.3	<0.00001
			9R:7S	1			0.1	0.7383*			12.9	0.0003
			7R:9S	1			17.9	<0.00001			56.1	<0.00001
			5R:11S	1			75.6	<0.00001			148.5	<0.00001
			11R:5S	1			14.5	0.0001			0.1	0.7469*
			13R:3S	1			89.8	<0.00001			28.5	<0.00001
			3R:13S	1			233.4	<0.00001			377.2	<0.00001
			15R:1S	1			541.3	<0.00001			275.0	<0.00001
			1R:15S	1			1063.9	<0.00001			1544.4	<0.00001
			63R:1S	1			2626.8	<0.00001			1460.2	<0.00001
			1R:63S	1			4831.2	<0.00001			6813.9	<0.00001

*Observed segregation ratio not significantly different from the tested ratio at P ≤ 0.05. ^ϕ^Pathotype 3H (SACAN-ss1) is a single spore isolate (Xue et al., 2008) while isolates represented by pathotypes 5X (LG-1) and 5G (CDCS) are field isolates (Strelkov et al., 2016, 2018). ^1^Plants with disease score = 0 as R and those with disease scores 1, 2 and 3 as S. ^2^Plants with disease scores = 0 and 1 as R and those with disease scores 2 and 3 as S.

Sixty-eight and 50 plants of two of the six *F*_2_ families from Popl#1 inoculated with pathotype 3H fit the 3R:1S segregation ratio in addition to the 9R:7S, 13R:3S and or 11R:5S ratios (Table 1). Thirty-four, 151 and 55 *F*_2_ plants of three families exhibited segregation ratios of 1R:3S, 9R:7S, 7R:9S, 5R:11S, and 3R:13S to pathotype 3H while 176 *F*_2_ plants of one family exhibited segregation ratios of 7R:9S and 9R:7S (Table 1). Seventy-six, 47, 142, 163, 56, and 40 plants of the six *F*_2_ families of Popl#1 inoculated with pathotype 5X showed segregation ratios of 1R:3S, 9R:7S, 7R:9S, 5R:11S, 3R:13S, 1R:15S, and 1R:63S (Table 1). Finally, all 490 (pooled data for the six *F*_2_ families) *F*_2_ plants of Popl#1 inoculated with pathotype 5G fit a 1R:3S and 5R:11S segregation ratio (Table 1). About 73.2% of the observed segregation ratios were 9R:7S, 7R:9S, 11R:5S, 5R:11S 13R:3S, 3R:13S, and 1R:15S.

In the case of Popl#2, the 227 *F*_2_ plants (derived from a single cross) inoculated with pathotype 3H gave a good fit for 9R:7S and 7R:9S (Table 2). Three ratios, 3R:1S, 9R:7S, and 11R:5S could fit the data obtained with the 244 *F*_2_ plants inoculated with pathotype 5X. Ratios of 9R:7S and 11R:5S segregation ratios could fit the data for the 239 *F*_2_ plants inoculated with pathotype 5G (Table 2). About 85.7% of the observed segregation ratios were 9R:7S, 7R:9S, and 11R:5S. Overall, 74.6% of the observed segregation ratios were deviations from 15R:1S segregation ratio expected if two major genes controlled clubroot resistance.

### Linkage Analyses and QTL Mapping

Of 144 PCR-based markers screened by bulk segregant analysis, 49 markers detected polymorphism between the parents of Popl#1 (i.e., ECD 02 and CR 2599), while 45 markers detected polymorphism between the parents of Popl#2 (i.e., ECD 02 and CR 1505). Twenty-seven of the 49 and 23 of the 45 markers detected polymorphisms in Popl#1 and Popl#2, respectively. Sixteen (14 from A03 + 2 from A08) of these markers detected polymorphism in the two *F*_2_ populations, while 11 and 7 markers detected polymorphism only in Pop#1 and Popl#2, respectively. Table 3 provides the sequence information and origin of the 34 (16 + 11 + 7) polymorphic markers used to genotype the two *F*_2_ populations.

**TABLE 3:** List of polymorphic markers used for linkage map construction and QTL analysis.

Marker	Chrom	Flanked/associated *CR* gene	Forward	Reverse	References
GC2360	A03	*CRa*	CAGCACCAGCATAACCAGCTACAGTC	AGAACTTTGCAAGTGGCTCAGATAAT	Matsumoto et al., 2012
GC2920	A03	*CRa*	CAAAGAACTGCCTGTTGTAAGTAAA	TGTTCAACAAGTTCCCATCTCCAT	Matsumoto et al., 2012
JY14	A03	*CRa*	GCGTGTTTGATGACTTTCCCT	GGTGGTGGAAACCCTAGGAA	This study
JY44	A03	*CRa*	AGACTTTGCAAGACCTCAACA	CTGAAGAGGAACAGGGTCAT	This study
CRaJY	A03	*CRa*	GTTGGAGACGGAGGTGAAGA	GCATCCCGTGAGATTCAGTT	This study
TCR05	A03	*CRb*	AGAATCATGACCGGGGAAAT	GCAGCTAAGTCATCGACCAA	Piao et al., 2004
TCR09	A03	*CRb*	GCAGCAACCGATAATATAAGGA	AACCAGAAGAAGAAAAACAAAAA	Piao et al., 2004
TCR17	A03	*CRb*	GCACATCACTTTGAGGACGA	TTTCCGTTGTCCTTTGTGAA	Piao et al., 2004
TCR30	A03	*CRb*	CGTGGATCTCGTCTTCAGGT	GGAACAGTATACTTCCCGGTGT	Zhang et al., 2014
TCR74	A03	*CRb*	ATGGATGATGGATGGATAGAGTG	TTGAACCATAGGAGGGATAGTTG	Zhang et al., 2014
TCR79	A03	*CRb*	TGACGTTCAATCAAAGCCTGA	TTTAGCAATCAAATGCAAATTCAA	Zhang et al., 2014
KBrH129J08Rc	A03	*CRb*^Kato^	ATGAGATTGAAGAGGGAAACACAA	GTTTCCAATGGTGAAACCAATCCTA	Kato et al., 2012
KBrH129J18R	A03	*CRb*^Kato^	AGAGCAGAGTGAAACCAGAACT	GTTTCAGTTCAGTCAGGTTTTTGCAG	Kato et al., 2012
KBrB091M11R	A03	*CRb*^Kato^	ACTTAAAGCACGAGAATGCAAA	GTTTGGTGTCGAAGCTATGTGTG	Kato et al., 2012
KB69N05	A03	*CRb*^Kato^	ATCACAACCAAAATGGAATGAC	GTTTCTCAAGCACCGAGACTCATAA	Kato et al., 2013
KB59N06	A03	*CRb*^Kato^	ATGAAATTGCAACTCTCAAAATG	GTTTAGGCTTTCTCCATCAACCACTA	Kato et al., 2013
KB59N03	A03	*CRb*^Kato^	AGGTAAATCCTCAAAAAGCCAT	GTTTGGCGAAATTCAGTTGACA	Kato et al., 2013
B4732	A03	*CRb*^Kato^	ATCTGATGTACCTTTGTGCTGG	GTTTGTCAATCATTCAAGCTAAGTGG	Kato et al., 2013
B0903	A03	*CRb*^Kato^	ACTTCCTCTGCTTTTCTCAGGT	GTTTGAAACTCTTCTCCCCCTTC	Kato et al., 2013
BGA06	A03	*CRb*^Kato^	AGAAATAGCAAAGCTCAAACGG	GTTTCCAGAAAAGAGATGCAGACAA	Kato et al., 2013
BGB41	A03	*CRb*^Kato^	ATCGCATAAACTAATAAAAATCAAAA	GTTTGACCCACATGATTAACAA	Kato et al., 2013
KB29N19	A03	*CRb*^Kato^	ATGAGATCGTCAGCCATTTCTC	GTTTCCAGTCCGGTTTTTATTACCTT	Kato et al., 2013
KB29N16	A03	*CRb*^Kato^	AGACTCGACAAGGTATCGATCT	GTTTGACGCCATTATGACACAACT	Kato et al., 2013
KB29N11	A03	*CRb*^Kato^	ACTCTCCACCAACACTTCCTAA	GTTTGAAGCTATCTTAGACCACC	Kato et al., 2013
KB91N13	A03	*CRb*^Kato^	AGACGGAGACTTTGAGATCTGG	GTTTCGAGTACTTCCAGAAACACG	Kato et al., 2013
Ol11-G11	A03	Crr3	GTTGCGGCGAAACAGAGAAG	GAGTAGGCGATCAAACCGAG	Fredua-Agyeman and Rahman, 2016
BrSTS-020	A03	*Crr3*	CTTCAGAACATCAGAAAGGGTCTT	TTGTTAATCTTGGTTGGGATGTTA	Fredua-Agyeman and Rahman, 2016
BRMS-050	A03	*CRb*	AACTTTGCTTCCACTGATTTTT	TTGCTTAACGCTAAATCCATAT	Fredua-Agyeman and Rahman, 2016
BnGMS344	A03	*CRb*	TGGGAAGAATCTCGTTAGAA	TCTCCTCTTCGGTTACGATA	Fredua-Agyeman and Rahman, 2016
Na10-B01	A03	*CRk*	CAAGTGTCTGCTAGGTGGGG	TCGATCGAAGAAACCAGACC	Fredua-Agyeman and Rahman, 2016
Ol11-B05	A03	*CRk*	TCGCGACGTTGTTTTGTTC	ACCATCTTCCTCGACCCTG	Fredua-Agyeman and Rahman, 2016
Na14-E02	A03	*CRk*	ACTGGCTACATGAGTTTCAGTG	GAGGGAAGACAACTGGTCTCA	Fredua-Agyeman and Rahman, 2016
BRMS-088	A08	*Crr1*	TATCGGTACTGATTCGCTCTTCAAC	ATCGGTTGTTATTTGAGAGCAGATT	Suwabe et al., 2003
A08-5021	A08	*Crr1*	CAGATGAGACAACACAGGAAACA	ACTCAATACGTTTTTCGCGG	Hasan and Rahman, 2016

**FIGURE 3:**
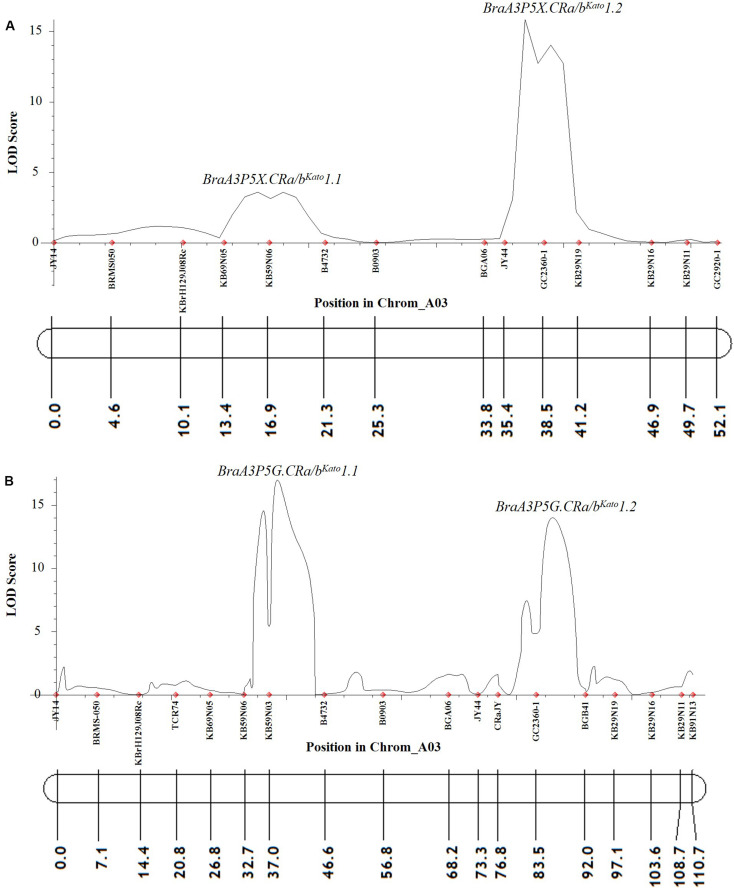
QTL likelihood profile and partial linkage map of the A03 chromosome of *Brassica napus* and *Brassica rapa* showing resistance to *P. brassicae* pathotypes 5X **(A)** and 5G **(B)** in Popl#1. The LOD scores are indicated on the *y*-axis while the marker names and the genetic distances (in cM) are indicated on the *x*-axis. Two QTLs were detected for resistance to pathotypes 5X **(A)** and 5G **(B)**. The first QTL (*BraA3P5X.CRa/b*^Kato^*1.1* and *BraA3P5G.CRa/b*^Kato^*1.1*) was detected in the genomic region where the *CRb*^Kato^ gene was mapped (Kato et al., 2012, 2013) while the second QTL (*BraA3P5X.CRa/b*^Kato^*1.2* and *BraA3P5G.CRa/b*^Kato^*1.2*) was detected in the genomic region where the *CRa* gene was mapped (Matsumoto et al., 1998, 2012; Hayashida et al., 2008; Ueno et al., 2012).

At a LOD threshold of 3.0 and a recombination fraction (O) of 0.40, 14 of the 25 A03 chromosome markers used to screen the *F*_2_ plants of Popl#1 inoculated with pathotype 5X were linked, while 11 of the A03 chromosome markers remained unlinked. Based on the ICIM-ADD method, two QTLs from the A03 chromosome of *B. napus* were detected for resistance to pathotype 5X. The SSR markers KB69N05 and B4732 flanked the first QTL *BraA3P5X.CRa/b*^Kato^*1.1* [LOD score = 3.6, located between 13.4 and 21.3 cM on Figure 3A and ≈24274312–24348056 base pairs (bp) on the physical map of *B. napus*]. The SSR markers BGA06 and KB29N19 bordered the second QTL *BraA3P5X.CRa/b*^Kato^*1.2* (LOD score = 15.9, located between 33.8–41.2 cM on Figure 3A and ≈24426905–24637310 bp on the physical map of *B. napus*). These two QTLs explained 4.5 and 51.0% of the phenotypic variance, respectively. Single Marker Analysis (SMA) by the IciMapping software showed that the SCAR marker GC2360-1 had the highest LOD and PVE (phenotypic variation explanation) scores of 14.2 and 48.9%, respectively, followed by marker KB59N06 with a LOD and PVE scores of 5.7 and 23.8%, respectively. Overall, the linked markers explained 55.4 and 72.7% of the phenotypic variance with the ICIM-ADD and SMA methods, respectively.

Similarly, in the *F*_2_ plants from Popl#1 inoculated with pathotype 5G, 18 of the 25 markers on the A03 chromosome were linked, while three of the markers remained unlinked. The ICIM-ADD method identified two QTLs from the A03 chromosome of *B. napus* for resistance to pathotype 5G. The SSR markers KB59N06 and B4732 bordered the first QTL *BraA3P5G.CRa/b*^Kato^*1.1* (LOD score = 17.0, located between 32.7–46.6 cM on Figure 3B and ≈24262454–24348056 bp on the physical map of *B. napus*). The SSR markers CRaJY and BGB41 flanked the second QTL *BraA3P5G.CRa/b*^Kato^*1.2* (LOD score = 14.0, located between 76.8–92.0 cM on Figure 3B and ≈ 24557499–24579679 bp on the physical map of *B. napus*). These two QTLs explained 23.3 and 21.8% of the phenotypic variance, respectively. The SMA by the IciMapping software showed that SSR marker KB59N03 had the most significant association with LOD and PVE scores of 6.8 and 27.4%, respectively. This was followed by marker GC2360-1 with LOD and PVE scores of 6.3 and 25.7%, respectively. Overall, based on the IciMapping software and using the ICIM-ADD and SMA methods, the PVE by the linked markers was 45.2 and 53.1%, respectively.

**FIGURE 4:**
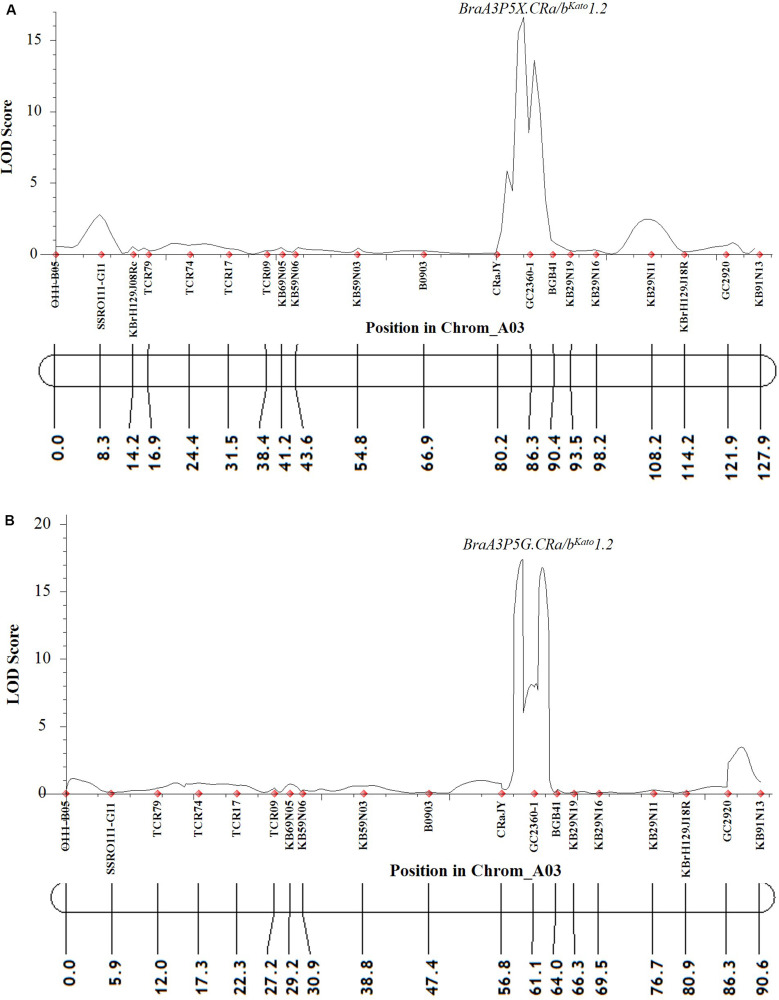
QTL likelihood profile partial linkage map of the A03 chromosome of *B. napus* and *B. rapa* showing resistance to two *P. brassicae* pathotypes 5X **(A)** and 5G **(B)** in Popl#2. Pathotypes 5X and 5G used in the inoculation experiments could overcome clubroot resistance in commercial canola cultivars (Strelkov et al., 2016, 2018). The LOD scores are indicated on the *y*-axis while the marker names and the genetic distances (in cM) are indicated on the *x*-axis. Only one QTL (*BraA3P5X.CRa/b*^Kato^*1.2* and *BraA3P5G.CRa/b*^Kato^*1.2*) was detected for resistance to pathotypes 5X **(A)** and 5G **(B)**. This QTL was detected in genomic regions of the A03 chromosome of *B. napus* and *B. rapa* where the *CRa*/*CRb*^Kato^ gene(s) was reported (Matsumoto et al., 1998, 2012; Hayashida et al., 2008; Kato et al., 2012, 2013; Ueno et al., 2012).

**FIGURE 5:**
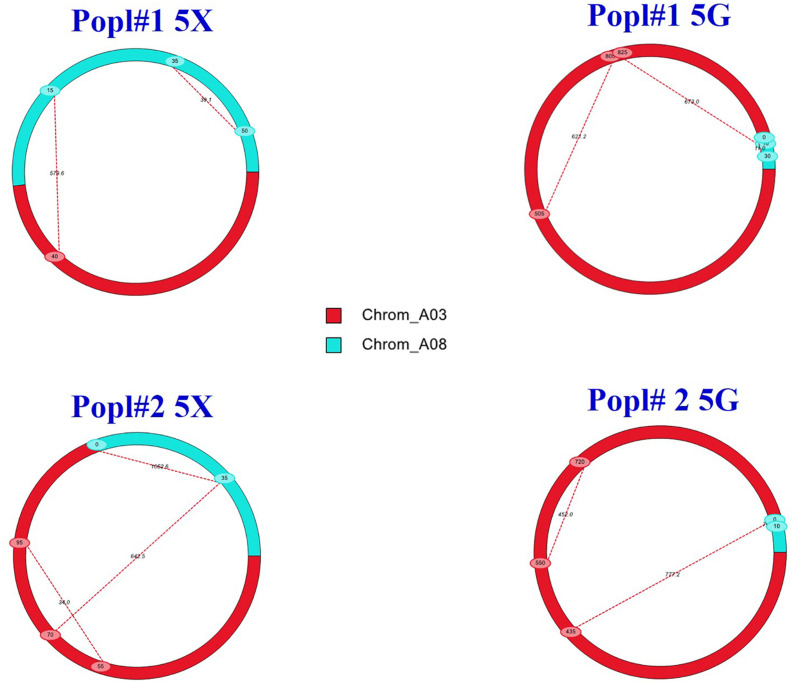
Epistatic QTLs conferring clubroot resistance in two *F*_2_ mapping populations (Popl#1 and Popl#2) as revealed by QTL IciMapping software V4.1. The dashed lines represent epistatic interaction pairs of CR QTLs. The red and cyan colors indicate A03 and A08 QTLs, respectively.

For Popl#2 *F*_2_ plants inoculated with pathotype 5X and 5G, the linkage analysis revealed that almost all (at least 19 of the 21) of the polymorphic markers on the A03 chromosome were linked. However, only the QTLs *BraA3P5X.CRa/b*^Kato^*1.2* and *BraA3P5G.CRa/b*^Kato^*1.2* were detected for resistance by the ICIM-ADD method in the case of the *F*_2_ plants of Popl#2 inoculated with pathotype 5X (LOD score = 16.7, Figure 4A) and 5G (LOD score = 17.4, Figure 4B), respectively. The QTL was located in the interval between SSR markers CRaJY and KB29N19 in the genomic region located approximately 24557499–24637310 bp on the physical map of *B. napus*. The percent of phenotypic variance explained by the ICIM-ADD method was 35.6 and 32.5% for the *F*_2_ plants inoculated with pathotype 5X and 5G, respectively. The SMA by the IciMapping software showed that SSR marker GC2360-1 had the most significant association in response to infection by *P. brassicae* pathotypes 5X and 5G. The LOD scores for the SMA was 6.6 and 4.7 for the *F*_2_ plants inoculated with pathotype 5X and 5G while the PVE scores was 27.0 and 29.3%, for pathotype 5X and 5G, respectively.

The LOD profiles and PVE determined by the CIM and SMA methods implemented in the WinQTL Cartographer software for the *F*_2_ plants from Popl#1 and Popl#2 inoculated with pathotypes 5X and 5G showed the same pattern as those determined with the IciMapping software (Supplementary Figures S1, S2). For example, the LOD score values for *BraA3P5X.CRa/b*^Kato^*1.1* and *BraA3P5X.CRa/b*^Kato^*1.2* for pathotype 5X were 4.2 and 8.2, respectively for the CIM method compared with 3.6 and 15.9 by the ICIM-ADD method. The LOD score values for *BraA3P5G.CRa/b*^Kato^*1.1* and *BraA3P5G.CRa/b*^Kato^*1.2* for pathotype 5G were 21.3 and 6.5, respectively for the CIM method compared with 17.0 and 14.0 by the ICIM-ADD method. In the case of Popl#2, the LOD scores for the QTLs *BraA3P5X.CRa/b*^Kato^*1.2* and *BraA3P5G.CRa/b*^Kato^*1.2* were 6.2 and 6.9 for pathotypes 5X and 5G, respectively, for the CIM method compared with 16.7 and 17.4 by the ICIM-ADD method. Thus, the LOD scores determined with the ICIM software were in general about two times higher than the values obtained with the WinQTL cartographer software.

Recombination between the markers KB59N06 (*BraA3P5X.CRa/b*^Kato^*1.1*) and KB59N03 (*BraA3P5G.CRa/b*^Kato^*1.1*) which had the most significant association with clubroot resistance in the *F*_2_ plants of Popl#1 inoculated with pathotypes 5X and 5G was 21.6 and 20.8%, respectively. Recombination in SSR marker GC2360-1(*BraA3P5X.CRa/b*^Kato^*1.2* and *BraA3P5G.CRa/b*^Kato^*1.2*), which showed the most significant association with clubroot resistance in the *F*_2_ plants of Popl#1 and Popl#2 inoculated with pathotypes 5X and 5G ranged from 11.5 to 18.8% and 12.5 to 22.9%, respectively. In the case of the two co-segregating markers on the A08 chromosome, recombination between the BRMS-088 allele and clubroot resistance in the *F*_2_ plants of Popl#1 inoculated with pathotype 5X and 5G was 10.4 and 15.6%, respectively. Similarly, recombination between marker allele and phenotype was 7.3 and 25.0% in the *F*_2_ plants of Popl#2 inoculated with pathotype 5X and 5G, respectively. Recombination between the SSR marker A08-5021 allele and clubroot resistance in the *F*_2_ plants of Popl#1 inoculated with pathotypes 5X and 5G was 9.4 and 28.1%, respectively, while the recombination in Popl#2 was 7.3 and 11.5%, respectively. Thus, the recombination between the two A08 markers and clubroot resistance to pathotype 5X was much smaller (7.3–10.4%) compared with that for resistance to pathotype 5G (11.5–28.1%).

The additive effects detected by the IciMapping and the WinQTL cartographer software were positive for the QTLs, which suggested that the alleles conferring clubroot resistance were derived from the resistant parent ECD 02. Epistatic interaction (Q × Q) analysis showed PVE at levels ranging from 0.0 to 17.6% for the A03 × A03 QTLs, 35.6–53.4% for the A03 × A08 QTLs and 25.8–37.3% for the A08 × A08 QTLs. The results of the epistatic QTL analysis (Figure 5) suggested that genes from both the A03 and the A08 chromosomes are needed for resistance to be effective.

## Discussion

Clubroot is widespread in many of the *Brassica* growing areas of the world. Both quantitative and qualitative disease resistance have been reported in *Brassica* crops (Piao et al., 2009). The erosion of single dominant CR genes in commercial canola cultivars and the emergence of new virulent isolates of *P. brassicae* have been reported in Canada (Strelkov et al., 2016, 2018) and Europe (Oxley, 2007; Diederichsen et al., 2014; Wallenhamar et al., 2014; Zamani-Noor, 2017). The erosion of the effectiveness of CR genes has also occurred in cruciferous vegetables in Asia (Bhattacharya et al., 2014; Chai et al., 2014). The elevated infection in clubroot-resistant cultivars and volunteers would lead to increased spore load of the pathogen in the soil (Zamani-Noor and Rodemann, 2017). Hence, clubroot remains a huge problem and poses by far the most significant threat to cruciferous crop production worldwide.

One of the strategies to combat clubroot caused by the many pathotypes of *P. brassicae* is to deploy cultivars with multiple CR genes. The inheritance of different encoding genes provides a buffer when one resistance mechanism is overcome (Lagudah, 2011). However, the combined effects of inheriting gene combinations seems to be very complex. In durum wheat (*Triticum turgidum* L. ssp. *durum*), strong additive effects of the *Lr34*/*Yr18* gene combination to leaf rust, stripe rust and powdery mildew were observed compared to the resistance effects of the *Lr46*/*Yr29* gene combination to the three diseases (Lillemo et al., 2008). Therefore, in canola different gene combinations are expected to confer different levels of resistance to clubroot.

In this study, the inheritance of multiple CR genes was examined in *F*_2_ plants derived from the crossing between *B. rapa* L. spp. *rapifera* line ECD 02 (turnip) (the resistant parent) and two *B. rapa* accessions (the susceptible parents). The fact that all the F_1_ plants from the above crosses were highly resistant to pathotypes 3H, 5X, and 5G suggested that the resistance gene in ECD 02 is dominant and could be due to the combined effect of two or more genes. The F_1_ means of all crosses were not significantly different from the resistant parent ECD 02 but were significantly different from the mean measurements of the two susceptible parents. These results indicate the complete dominance genes controlling clubroot resistance genes came from ECD 02. The segregation of *F*_2_ plants for clubroot resistance (Tables 1, 2) and the fact that the *F*_2_ means of all crosses were significantly different from the mean measurements of both parents suggested that the genetic variance consisted of both additive and dominance effects. The 3R:1S and 1R:3S ratios are consistent with the inheritance of a trait controlled by a single dominant major gene. Digenic dominant epistasis, represented as 12R:4S and 4R:12S, gives the same 3R:1S and 1R:3S ratios, respectively. Therefore, based on 3R:1S/1R:3S ratios, we could not conclude whether there was one or two CR genes in ECD 02. On the other hand, the 9R:7S, 7R:9S, 5R:11S, 11R:5S, 13R:3S, 3R:13S, and 1R:15S ratios are modifications of the 15R:1S segregation ratio expected for a trait controlled by two dominant major genes.

The distorted segregation ratios suggested that the resistance genes were on different chromosomes (Hayman and Mather, 1955). The 9R:7S ratio confirms the existence of two genes with duplicate recessive epistasis, which suggested that the dominant allele at the two loci were necessary to control the clubroot caused by pathotypes 5X and 5G. In other words, individuals with double recessive at either locus or both loci were susceptible. The 13R:3S ratio shows recessive suppression in which the presence of two recessive alleles of one gene are needed to suppress the effects of the dominant gene. The 5R:11S, 11R:5S, 7R:9S, 3R:13S, and 1R:15S ratios indicated two-gene control of clubroot resistance with digenic additive epistasis or quantitative control of the resistance. In the current study, most of the segregation ratios fit the two-gene model although segregation patterns of 3R:1S, 1R:3S, and 1R:63S were also possible. However, the mapping population was of the *F*_2_ generation and hence it is not clear if the preponderance of non-allelic gene effects will alter at advanced generations. Pioneering studies based on segregation ratios suggested that at least two dominant major genes (originally designated A and C) controlled clubroot resistance in ECD 02 (Wit and Van De, 1964; Buczacki et al., 1975). Therefore, the results of the greenhouse screening work was in agreement with the published literature.

European turnips are a very rich source of dominant major CR genes and hence have been used as resistance donors in many breeding programs around the world. Published CR genes derived from turnips include *Crr1*, *Crr2*, *Crr3*, *Crr4*, *CRa*, *CRb*, *CRb*^Kato^, *CRc*, *CRd*, *CRk*, *Rcr1*, *BraA.Cr.a*, *BraA.Cr.b*, *BraA.Cr.c*, and *CrrA5* (Matsumoto et al., 1998, 2012; Suwabe et al., 2003, 2006; Hirai et al., 2004; Piao et al., 2004; Hirai, 2006; Saito et al., 2006; Sakamoto et al., 2008; Kato et al., 2012, 2013; Ueno et al., 2012; Hatakeyama et al., 2013; Chu et al., 2014; Zhang et al., 2014; Yu et al., 2016; Hirani et al., 2018; Nguyen et al., 2018; Pang et al., 2018). Most of the previous genetic mapping studies in Canada and Europe relied on single gene resistance resources as donors (Diederichsen et al., 2009; Chu et al., 2014; Fredua-Agyeman and Rahman, 2016; Hasan and Rahman, 2016; Yu et al., 2016). Matsumoto et al. (1998) reported that ECD 02 possessed the *CRa* gene. Ueno et al. (2012) mapped the *CRa* gene to a genomic region on the A03 chromosome of *B. rapa* between the SCAR markers GC2360 and GC1680. Hirani et al. (2018) reported that ECD 02 possessed another clubroot resistance gene, *BraA.CR.b*, on the A08 chromosome of *B. rapa* that could be similar to the *Crr1* gene previously reported by Hatakeyama et al. (2013). Therefore, the genomic regions identified in this study was consistent with the results of previous studies that mapped the clubroot resistance loci derived from ECD 02 to the A03 and the A08 chromosomes.

In spite of the substantial contribution of non-additive effects to the variation of complex traits, gene effects controlling clubroot resistance have not been studied. Yu et al. (2017) reported that the *Rcr8* gene on the A02 chromosome and *Rcr9* gene on the A08 chromosome conferred resistance to pathotype 5X. However, the induced resistance to pathotype 5X was not correlated with the resistance to pathotypes 2F, 3H, 5I, 6M, and 8N conferred by the *Rcr4* gene on the A02 chromosome (Yu et al., 2017). On the other hand, our study shows that resistance conferred by the *CRa*/*CRb*^Kato^ gene(s) on the A03 and the *Crr1* gene on the A08 chromosome also conferred resistance to pathotype 3H. In addition, Yu et al. (2017) did not show the different interactions involving the multiple (*Rcr4*, *Rcr8*, and *Rcr9*) resistance genes they identified. To the best of our knowledge, our study is the first report that demonstrates that the two CR genes in ECD 02 interact in a non-additive manner to confer resistance to clubroot. Such non-allelic interactions of multiple genes have been reported in the response of many plants to fungi, bacteria, virus and insect attack. For example, barrel clover (*Medicago truncatula*) to aphid attack (Kamphius et al., 2020); mungbean (*Vigna radiata*) to mungbean yellow mosaic virus attack (Akbar et al., 2017); soybean (*Glycine max*) to rust infection (Pierozzi et al., 2008) and cotton (*Gossypium hirsutum*) to insect pest and virus attacks (Ahuja et al., 2007).

In summary, clubroot tests, linkage analysis and QTL mapping carried out in this study demonstrated that the *CRa/CRb*^Kato^ and the *Crr1* genes on the A03 and the A08 chromosomes of ECD 02 interact in a non-allelic manner to confer resistance to pathotypes 5X and 5G. Based on the QTL analysis, the genetic control against virulent *P. brassicae* pathotypes may also involve additional genes modulating the action of the two major genes. The presence of at least the two dominant genes complementing each other might explain why ECD 02 confers strong and highly stable qualitative resistance to many *P. brassicae* pathotypes from around the world. Knowledge of gene effects controlling clubroot resistance offers the possibility of exploiting ECD 02 resistance for the breeding of clubroot resistant canola cultivars and cruciferous vegetables. In addition, the genomic regions identified in this study will provide additional resources for marker-assisted selection in *Brassica* breeding programs.

## Data Availability Statement

All datasets generated for this study are included in the article/Supplementary Material.

## Author Contributions

RF-A: conceptualization, grant application, genetic crosses to produce F_1_ seeds, screening of F_1_ plants, development of *F*_2_ mapping populations, PCR, genotyping, data analysis, and writing of the manuscript. JJ: screening of F_1_ plants, development of *F*_2_ mapping populations, inoculum preparation, screening of *F*_2_ plants, disease rating, DNA extraction, PCR, genotyping, and writing the basic draft of manuscript. S-FH: grant application, supervision and provision of technical support for all pathotyping work carried in the greenhouse, and revision of the manuscript. SS: grant application, supervision and provision of *Plasmodiophora brassicae* pathotypes, pathotyping work for clubroot, and revision of manuscript. All authors contributed to the article and approved the submitted version.

## Conflict of Interest

The authors declare that the research was conducted in the absence of any commercial or financial relationships that could be construed as a potential conflict of interest.
