# Supplementary material for: QTL Mapping and Inheritance of Clubroot Resistance Genes Derived From Brassica rapa subsp. rapifera (ECD 02) Reveals Resistance Loci and Distorted Segregation Ratios in Two F2 Populations of Different Crosses
Source: Front Plant Sci. 2020 Jul 3;11:899. doi: 10.3389/fpls.2020.00899 (PMC7348664; doi:10.3389/fpls.2020.00899)
Supplement: Supplementary file 1 [file Data_Sheet_1.zip › Table S1.docx]

**Table S1.** Clubroot tests of F_1_ plants of ECD 02* × [CR 2599^α^ and CR 1505^β^]

| F_1_ plants of the cross | *P. brassicae*  Pathotype^a^ | Disease score^b^ | | | |
| --- | --- | --- | --- | --- | --- |
|  |  | 0 | 1 | 2 | 3 |
| ECD 02* × CR 2599^α^ | 2F | 2 | 0 | 0 | 0 |
|  | 3H | 2 | 0 | 0 | 0 |
|  | 5I | 0 | 2 | 0 | 0 |
|  | 6M | 3 | 0 | 0 | 0 |
|  | 8N | 3 | 0 | 0 | 0 |
|  | 2B | 1 | 0 | 0 | 0 |
|  | 5X (L-G1) | 4 | 0 | 0 | 0 |
|  | 5G | 4 | 1 | 0 | 0 |
|  | 8J | 2 | 1 | 0 | 0 |
|  | **All pathotypes** | **21** | **4** | **0** | **0** |
| ECD 02* × CR 1505^β^ | 5X (L-G1) | 1 | 0 | 0 | 0 |
|  | 5G | 1 | 0 | 0 | 0 |
|  | **All pathotypes** | **2** | **0** | **0** | **0** |
| **ECD 02* × [CR 2599^α^ and CR 1505^β^]** | **Overall** | **23** | **4** | **0** | **0** |

* The resistant parent ECD 02 is *B. rapa* L. subsp. *rapifera* line AAbbCC.

^α, β^ The susceptible parents CR2599 and CR 1505 (cv. ‘Emma’) are *B. rapa* accessions.

^a^ Numbers beneath the scores represent the number of F_1_ plants evaluated for clubroot 8 weeks after inoculation. CR is clubroot resistant.

^b^ Pathotypes 2F, 3H, 5I, 6M and 8N are single-spore isolates identified prior to the introduction of CR varieties in Canada, while pathotypes 2B, 5X, 5G and 8J are field isolates identified after the introduction of CR varieties in Canada.
