# Supplementary material for: QTL Mapping and Inheritance of Clubroot Resistance Genes Derived From Brassica rapa subsp. rapifera (ECD 02) Reveals Resistance Loci and Distorted Segregation Ratios in Two F2 Populations of Different Crosses
Source: Front Plant Sci. 2020 Jul 3;11:899. doi: 10.3389/fpls.2020.00899 (PMC7348664; doi:10.3389/fpls.2020.00899)
Supplement: Supplementary file 1 [file Data_Sheet_1.zip › Table S2.docx]

**Table S2a.** Chi-square tests of homogeneity: F_2_ popl#1 screened with pathotype 3H (R = disease score 0; S= disease score 1 + 2 + 3).

| F_2_ family | No. of F_2_ plants with disease score | | | | Total | Obs R  (0) | Obs S  (1+2+3) | Exp R | Exp S | ((O-E)^2)  /E | χ^2^ | DF | Prob |
| --- | --- | --- | --- | --- | --- | --- | --- | --- | --- | --- | --- | --- | --- |
|  | 0 | 1 | 2 | 3 |  |  |  |  |  |  |  |  |  |
| 1 | 10 | 4 | 2 | 18 | 34 | 10 | 24 | 13.2 | 20.8 | 0.77 | 37.20 | 5 | < 0.00001* |
| 2 | 39 | 18 | 2 | 9 | 68 | 39 | 29 | 26.4 | 41.6 | 6.06 |  |  |  |
| 3 | 34 | 4 | 11 | 102 | 151 | 34 | 117 | 58.5 | 92.5 | 10.28 |  |  |  |
| 4 | 65 | 12 | 9 | 90 | 176 | 65 | 111 | 68.2 | 107.8 | 0.15 |  |  |  |
| 5 | 39 | 1 | 0 | 10 | 50 | 39 | 11 | 19.4 | 30.6 | 19.86 |  |  |  |
| 6 | 20 | 7 | 3 | 25 | 55 | 20 | 35 | 21.3 | 33.7 | 0.08 |  |  |  |

**Table S2b.** Chi-square tests of homogeneity: F_2_ popl#1 screened with pathotype 3H (R = disease score 0 +1; S= disease score 2 + 3).

| F_2_ family | No. of F_2_ plants with disease score | | | | Total | Obs R  (0 + 1) | Obs S  (2+3) | Exp R | Exp S | ((O-E)^2)  /E | χ^2^ | DF | Prob |
| --- | --- | --- | --- | --- | --- | --- | --- | --- | --- | --- | --- | --- | --- |
|  | 0 | 1 | 2 | 3 |  |  |  |  |  |  |  |  |  |
| 1 | 10 | 4 | 2 | 18 | 34 | 14 | 20 | 16.1 | 17.9 | 0.28 | 46.82 | 5 | < 0.00001* |
| 2 | 39 | 18 | 2 | 9 | 68 | 57 | 11 | 32.2 | 35.8 | 19.06 |  |  |  |
| 3 | 34 | 4 | 11 | 102 | 151 | 38 | 113 | 71.5 | 79.5 | 15.73 |  |  |  |
| 4 | 65 | 12 | 9 | 90 | 176 | 77 | 99 | 83.4 | 92.6 | 0.49 |  |  |  |
| 5 | 39 | 1 | 0 | 10 | 50 | 40 | 10 | 23.7 | 26.3 | 11.23 |  |  |  |
| 6 | 20 | 7 | 3 | 25 | 55 | 27 | 28 | 26.1 | 28.9 | 0.03 |  |  |  |

* Phenotypic data of the different crosses are significantly different at *P* ≤ 0.05 and hence the data was not pooled before analysis.
