# Supplementary material for: QTL Mapping and Inheritance of Clubroot Resistance Genes Derived From Brassica rapa subsp. rapifera (ECD 02) Reveals Resistance Loci and Distorted Segregation Ratios in Two F2 Populations of Different Crosses
Source: Front Plant Sci. 2020 Jul 3;11:899. doi: 10.3389/fpls.2020.00899 (PMC7348664; doi:10.3389/fpls.2020.00899)
Supplement: Supplementary file 1 [file Data_Sheet_1.zip › Table S4.docx]

**Table S4a.** Chi-square tests of homogeneity: F_2_ popl#1 screened with pathotype 5G (R = disease score 0; S= disease score 1 + 2 + 3).

| F_2_ family | No. of F_2_ plants with disease score | | | | Total | Obs R  (0) | Obs S  (1+2+3) | Exp R | Exp S | ((O-E)^2)  /E | χ^2^ | DF | Prob |
| --- | --- | --- | --- | --- | --- | --- | --- | --- | --- | --- | --- | --- | --- |
|  | 0 | 1 | 2 | 3 |  |  |  |  |  |  |  |  |  |
| 1 | 8 | 4 | 0 | 13 | 25 | 8 | 17 | 6.3 | 18.7 | 0.44 | 2.24 | 5 | 0.8146 |
| 2 | 8 | 6 | 6 | 27 | 47 | 8 | 39 | 11.9 | 35.1 | 1.27 |  |  |  |
| 3 | 42 | 12 | 8 | 88 | 150 | 42 | 108 | 38.0 | 112.0 | 0.43 |  |  |  |
| 4 | 38 | 11 | 12 | 95 | 156 | 38 | 118 | 39.5 | 116.5 | 0.06 |  |  |  |
| 5 | 12 | 2 | 3 | 33 | 50 | 12 | 38 | 12.7 | 37.3 | 0.03 |  |  |  |
| 6 | 16 | 5 | 4 | 37 | 62 | 16 | 46 | 15.7 | 46.3 | 0.01 |  |  |  |

**Table S4b.** Chi-square tests of homogeneity: F_2_ popl#1 screened with pathotype 5G (R = disease score 0 +1; S= disease score 2 + 3).

| F_2_ family | No. of F_2_ plants with disease score | | | | Total | Obs R  (0 + 1) | Obs S  (2+3) | Exp R | Exp S | ((O-E)^2)  /E | χ^2^ | DF | Prob |
| --- | --- | --- | --- | --- | --- | --- | --- | --- | --- | --- | --- | --- | --- |
|  | 0 | 1 | 2 | 3 |  |  |  |  |  |  |  |  |  |
| 1 | 8 | 4 | 0 | 13 | 25 | 12 | 13 | 8.4 | 16.6 | 1.58 | 2.70 | 5 | 0.7458 |
| 2 | 8 | 6 | 6 | 27 | 47 | 14 | 33 | 15.7 | 31.3 | 0.19 |  |  |  |
| 3 | 42 | 12 | 8 | 88 | 150 | 54 | 96 | 50.2 | 99.8 | 0.29 |  |  |  |
| 4 | 38 | 11 | 12 | 95 | 156 | 49 | 107 | 52.2 | 103.8 | 0.20 |  |  |  |
| 5 | 12 | 2 | 3 | 33 | 50 | 14 | 36 | 16.7 | 33.3 | 0.45 |  |  |  |
| 6 | 16 | 5 | 4 | 37 | 62 | 21 | 41 | 20.8 | 41.2 | 0.00 |  |  |  |

* Phenotypic data of the different crosses are not significantly different at *P* ≤ 0.05 and hence data was pooled before analysis.
