# Supplementary figures and images for: QTL Mapping and Inheritance of Clubroot Resistance Genes Derived From Brassica rapa subsp. rapifera (ECD 02) Reveals Resistance Loci and Distorted Segregation Ratios in Two F2 Populations of Different Crosses
Source: Front Plant Sci. 2020 Jul 3;11:899. doi: 10.3389/fpls.2020.00899 (PMC7348664; doi:10.3389/fpls.2020.00899)

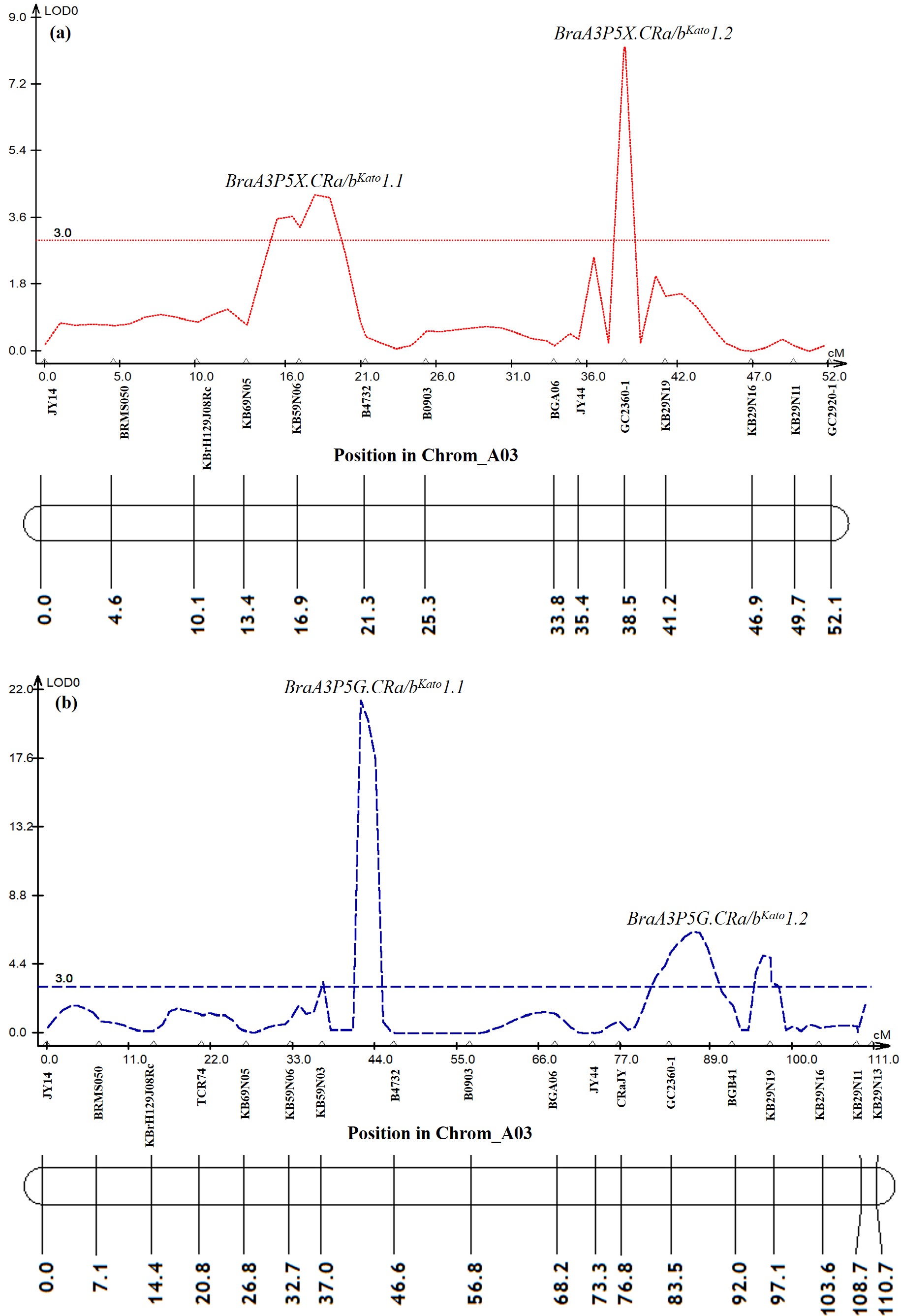

Supplement: Supplementary file 1 [file Data_Sheet_1.zip › Image 1.JPEG]

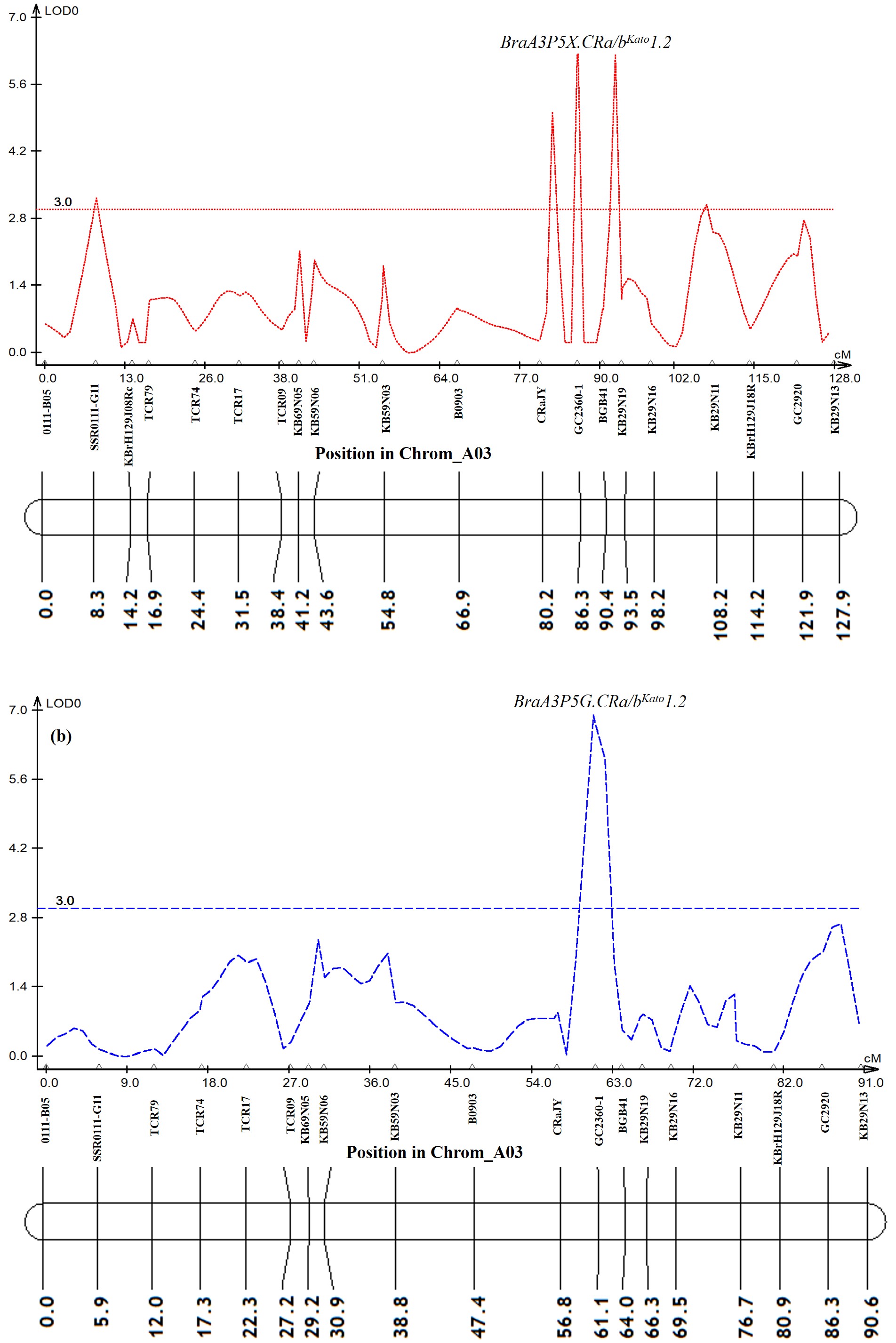

Supplement: Supplementary file 1 [file Data_Sheet_1.zip › Image 2.JPEG]
